# Associations of depression and regional brain structure across the adult lifespan: Pooled analyses of six population-based and two clinical cohort studies in the European Lifebrain consortium

**DOI:** 10.1016/j.nicl.2022.103180

**Published:** 2022-09-05

**Authors:** Julia Binnewies, Laura Nawijn, Andreas M. Brandmaier, William F.C. Baaré, David Bartrés-Faz, Christian A. Drevon, Sandra Düzel, Anders M. Fjell, Laura K.M. Han, Ethan Knights, Ulman Lindenberger, Yuri Milaneschi, Athanasia M. Mowinckel, Lars Nyberg, Anna Plachti, Kathrine Skak Madsen, Cristina Solé-Padullés, Sana Suri, Kristine B. Walhovd, Enikő Zsoldos, Klaus P. Ebmeier, Brenda W.J.H. Penninx

**Affiliations:** a Amsterdam UMC Location Vrije Universiteit Amsterdam, Department of Psychiatry, Amsterdam Neuroscience, Mood, Anxiety, Psychosis, Sleep & Stress Program, Amsterdam, The Netherlands; b Center for Lifespan Psychology, Max Planck Institute for Human Development, Berlin, Germany; c Max Planck, UCL Centre for Computational Psychiatry and Ageing Research, Berlin, Germany; d Department of Psychology, MSB Medical School Berlin, Berlin, Germany; e Danish Research Centre for Magnetic Resonance, Centre for Functional and Diagnostic Imaging and Research, Copenhagen University Hospital – Amager and Hvidovre, Copenhagen, Denmark; f Departament de Medicina, Facultat de Medicina i Ciències de la Salut, Universitat de Barcelona and Institut de Neurociències, Universitat de Barcelona, Spain; g Department of Nutrition, Institute of Basic Medical Sciences, Faculty of Medicine, University of Oslo & Vitas Ltd, Oslo Science Park, Oslo, Norway; h Center for Lifespan Changes in Brain and Cognition, University of Oslo, Norway; i Department of Radiology and Nuclear Medicine, Oslo University Hospital, Norway; j Centre for Youth Mental Health, The University of Melbourne, Parkville, VIC, Australia; k MRC Cognition and Brain Sciences Unit, University of Cambridge, Cambridge, United Kingdom; l Umeå Center for Functional Brain Imaging, Umeå University, Umeå, Sweden; m Radiography, Department of Technology, University College Copenhagen, Copenhagen, Denmark; n Wellcome Centre for Integrative Neuroimaging, University of Oxford, United Kingdom; o Department of Psychiatry, University of Oxford, United Kingdom

**Keywords:** Neuroimaging, Depressive symptoms, Meta-analysis, Lifespan, Grey matter, Brain structure

## Abstract

**Objective:**

Major depressive disorder has been associated with lower prefrontal thickness and hippocampal volume, but it is unknown whether this association also holds for depressive symptoms in the general population. We investigated associations of depressive symptoms and depression status with brain structures across population-based and patient-control cohorts, and explored whether these associations are similar over the lifespan and across sexes.

**Methods:**

We included 3,447 participants aged 18–89 years from six population-based and two clinical patient-control cohorts of the European Lifebrain consortium. Cross-sectional meta-analyses using individual person data were performed for associations of depressive symptoms and depression status with FreeSurfer-derived thickness of bilateral rostral anterior cingulate cortex (rACC) and medial orbitofrontal cortex (mOFC), and hippocampal and total grey matter volume (GMV), separately for population-based and clinical cohorts.

**Results:**

Across patient-control cohorts, depressive symptoms and presence of mild-to-severe depression were associated with lower mOFC thickness (r_symptoms_ = −0.15/ r_status_ = −0.22), rACC thickness (r_symptoms_ = −0.20/ r_status_ = −0.25), hippocampal volume (r_symptoms_ = −0.13/ r_status_ = 0.13) and total GMV (r_symptoms_ = −0.21/ r_status_ = −0.25). Effect sizes were slightly larger for presence of moderate-to-severe depression. Associations were similar across age groups and sex. Across population-based cohorts, no associations between depression and brain structures were observed.

**Conclusions:**

Fitting with previous meta-analyses, depressive symptoms and depression status were associated with lower mOFC, rACC thickness, and hippocampal and total grey matter volume in clinical patient-control cohorts, although effect sizes were small. The absence of consistent associations in population-based cohorts with mostly mild depressive symptoms, suggests that significantly lower thickness and volume of the studied brain structures are only detectable in clinical populations with more severe depressive symptoms.

## Highlights

• This meta-analysis included 3,447 participants from population and clinical cohorts.
• Depression associated with mOFC and rACC thickness, hippocampal volume and total GMV.
• Associations limited to clinical populations with more severe depressive symptoms.
• No associations with milder depressive symptoms of population-based cohorts.
• Consistent associations across the adult lifespan and between the sexes.

## 1 Introduction

Large meta-analyses pooling cohorts including clinically depressed patients and healthy controls, relate clinical depression in adults to a smaller grey matter structures in several regions, with most consistent findings and largest effect sizes for thickness of the rostral anterior cingulate (rACC), medial orbitofrontal cortex (mOFC) and hippocampal volume (Arnone et al., 2016, Schmaal et al., 2016, Schmaal et al., 2017), although effect sizes are modest (Cohen’s d −0.09 to −0.14). Whereas clinical depression is not uncommon with a prevalence of around 4.4 % (World Health Organization, 2017), mild depressive symptoms not reaching psychiatric thresholds are more common, with an estimated prevalence of up to 17 % of the general population (Rodríguez et al., 2012, Luppa et al., 2012). Due to this high prevalence, the burden of (mild) depression on society is high. Mild depressive symptoms are related to reduced quality of life (Rodríguez et al., 2012), increased disability burden (Barry et al., 2009), decreased physical health (Braam et al., 2005), increased economic costs (Luppa et al., 2012), and mortality risk (Win et al., 2011). Presence of mild depression also increases the risk of developing clinical depression (Cuijpers and Smit, 2004), leading to an even larger burden on the person and society (Cuijpers et al., 2014, Kessler, 2012, Hare et al., 2014). Some studies suggest that the smaller grey matter volumes in clinical depression are also present in individuals with mild depressive symptoms (Besteher et al., 2020). Whereas some studies report similar lower volume or thickness in the anterior cingulate (Webb et al., 2014, Hayakawa et al., 2014), orbitofrontal cortex (Webb et al., 2014) and hippocampus (Osler et al., 2018, Szymkowicz et al., 2019) for mild depression, others do not find these associations (Szymkowicz et al., 2016). However, no studies have investigated depressive symptoms and depression status in population-based cohorts as well as clinical patient-control cohorts.

A potential explanation for these inconsistencies might be small sample sizes, heterogeneity of applied methods and limited age ranges across studies. Grey matter generally decreases with age, with large variability across brain regions in the shape of these age-related changes across the lifespan (Pomponio et al., 2020, Walhovd et al., 2011). While total grey matter volume decreases rapidly during adolescence and early adulthood, followed by a slower reduction in adulthood, hippocampal volume only starts decreasing markedly at around 50 years of age (Pomponio et al., 2020). However, it is unclear how age may affect associations between depression and brain structure. Within the ENIGMA consortium, age did not affect the association between depression and regional cortical or subcortical brain structural measures but associations between cortical thickness and depression were only observed in adults and not in adolescents, who only displayed smaller surface area (Schmaal et al., 2016, Schmaal et al., 2017). Some studies reported more pronounced differences in brain structure in late-onset depression (Hickie et al., 2005, Sachs-Ericsson et al., 2013) while others found no associations of brain structure with late-life depression (Saberi et al., 2022). Thus, it is relevant to further investigate the moderating effect of age on associations between depression and brain structure, particularly across the adult lifespan.

Another factor potentially influencing associations between depression and brain structure is sex, whereby depression is much more prevalent in women than in men (Seedat et al., 2009). While in ENIGMA sex did not affect the association between depression and cortical measures (Schmaal et al., 2017), in another study depressive symptoms were only related to decreased anterior cingulate cortex volume in women but not in men (Hayakawa et al., 2014).

### 1.1 Aims of the study

We investigated the associations of depressive symptom severity and depression status with brain structure using data from eight neuroimaging cohorts across the adult lifespan (N = 3,449, 18–89 years). We focused on brain regions often associated with depression (bilateral mOFC, rACC, hippocampus) and expected more severe depressive symptoms and depression status to be associated with lower thickness and volumes. Additionally, total grey matter volume (GMV) was included as control measure of global brain structure for which no associations are expected. All analyses are conducted separately across population-based and patient-control cohorts, and across age and sex strata.

## 2 Material and methods

### 2.1 Sample

Adult participants (18 years or older) from the European Lifebrain consortium (Walhovd et al., 2018) (https://www.lifebrain.uio.no/) with available data on depressive symptoms and MRI measures were included. Samples included participants from six population-based cohorts: the Berlin Study of Aging-II (BASE-II, Germany) (Bertram et al., 2014), BETULA (Sweden) (Nilsson et al., 1997), the Cambridge Centre for Ageing and Neuroscience study (Cam-CAN, UK) (Shafto et al., 2014), Center for Lifebrain Changes in Brain and Cognition longitudinal studies (LCBC, Norway) (Walhovd et al., 2016, Fjell et al., 2018), Walnuts and Healthy Aging Study (WAHA, Spain) (Rajaram et al., 2017), Whitehall-II Imaging Sub-study (UK) (Filippini et al., 2014), and two cohorts including depressed patients and healthy controls (patient-control cohort): Netherlands Study of Depression and Anxiety (NESDA, Netherlands) (Penninx et al., 2008) and MOod Treatment with Antidepressants or Running study (MOTAR, Netherlands) (Lever-Van Milligen et al., 2019). Additional information for all cohorts and inclusion/exclusion criteria are presented in the Supplement.

### 2.2 Measurements

#### 2.2.1 Depression

Different depression instruments were used across cohorts: Beck Depression Inventory (BDI (Beck and Steer, 1993); depression cut-offs based on Wahl (Wahl et al., 2014)) for LCBC; 20-item Center for Epidemiological Studies-Depression Scale (CES-D (Radloff, 1977); cut-off for mild depression based on Radloff (Radloff, 1977), for moderate depression on Wahl (Wahl et al., 2014)) for BASE-II, Betula and Whitehall-II; Hamilton Depression Rating Scale (HDRS (Williams, 1988); cut-offs based on Zimmerman (Zimmerman et al., 2013)) for WAHA; Hospital Anxiety and Depression Scale (HADS (Zigmond and Snaith, 1983); cut-offs based on Zigmond and Snaith, 1983) for Cam-CAN; 30-item Inventory of Depressive Symptomatology - Self Report (IDS-SR (Rush et al., 1996); cut-offs based on Rush et al., 1996) for NESDA and MOTAR. All depression instruments were based on self-report, with the exception of the HDRS, which is clinician-rated. For each scale, the total score was used for analysis, and categorical variables were calculated based on validated and commonly used thresholds for at least mild (mild-to-severe) and at least moderate (moderate-to-severe) depression (see Table S1 for thresholds for each depression scale and Fig. S1 for distributions of depressive symptoms for each cohort).

#### 2.2.2 Imaging acquisition and analysis

Average thickness of the bilateral rostral anterior cingulate cortex (rACC) and medial orbitofrontal cortex (mOFC), hippocampal volume, total grey matter volume (GMV) and intracranial volume based on eTIV (ICV) were derived from T1 structural MRI scans using Freesurfer (version 5.3 for Whitehall-II, 6.0 for WAHA, LCBC, Cam-CAN, Betula, NESDA and MOTAR, and 7.0 for BASE-II). More details on scanner and MR acquisition parameters can be found in the Supplement (Table S1 and cohort descriptions).

### 2.3 Statistical analyses

All statistical analyses were conducted in R (version 3.6.0). Unadjusted brain structure data more than four standard deviations from the mean was excluded to remove outliers (N = 2). For analyses on depressive symptom scores with brain structure (i.e., thickness of rACC, mOFC, hippocampal volume, and total GMV), Spearman rank-order correlations were run for each cohort, corrected for age, sex, scanner, and ICV (the latter only for analyses of hippocampal volume and total GMV). Additional analyses were performed on depressive symptoms while also correcting for years of education to rule out potential differences in education to be driving the associations. To check whether the use of different Freesurfer versions across cohorts influenced results, meta-regressions of Freesurfer version were performed. To explore associations of depression status with the included brain structures, additional point biserial correlations were run on a dichotomized measure of depression (mild-to-severe vs not depressed), correcting for the same covariates. P-values and 95 % confidence intervals (CI) were calculated using bootstrap procedures with 5000 iterations. Depression status was only analysed in cohorts with at least 20 participants with mild-to-severe depression. The correlation estimates and CI’s from the per-cohort analyses were then pooled using the R package metafor (Viechtbauer, 2010) to obtain pooled estimates, using random-effects models. To examine effects in general population cohorts and as well as patient-control cohorts, meta-analyses were conducted separately for population-based cohorts and for patient-control cohorts. All statistical tests were performed two-sided and corrected for multiple testing using Bonferroni correction for four brain regions (α = 0.0125).

To explore effects of depression status severity, we performed additional sensitivity analyses on only a subgroup of the participants with moderate-to-severe depression in which we excluded participants with mild depression. This extreme comparison between participants with moderate-to-severe depression and participants without depression might be more comparable to comparing healthy controls to clinically depressed patients in clinical studies, as mild depressive symptoms might not warrant clinical diagnosis of depression. To test whether associations varied over the adult lifespan and between sexes, analyses of depression symptom scores were repeated with cohorts stratified by age (18–39 years, 40–59 years, 60 years and older), with only cohorts including at least 20 participants, and stratified by sex (without correction for age/ sex respectively). Forest plots were visually examined for consistently different patterns across age and sex bins. Additional meta-regressions were performed on proportion of women included in each cohort.

## 3 Results

### 3.1 Sample description

Descriptive statistics of the cohorts included in the meta-analyses are presented in Table 1. Eight cohorts from seven sites were included, of which six cohorts were population-based and two included clinically depressed patients and healthy controls (patient-control cohorts). In total 3449 participants were included, 3042 from population-based cohorts and 407 from patient-control cohorts. From the population-based cohorts, 287 (9 %) participants met the criteria for at least mild depression, of whom 69 (2 %) met the criteria for at least moderate depression. From the patient-control cohorts, 253 (62 %) met the criteria for at least mild depression, of which 193 (47 %) had at least moderate depression. The age range across all cohorts was 18 to 89 years (see supplemental Fig. 1 for age distributions of all cohorts).

**Table 1:** Demographic characteristics per cohort.

	**Population-based cohorts**						**Patient-control cohorts**	
	Whitehall-II (n = 769)	WAHA (n = 120)	LCBC (n = 742)	Cam-CAN (n = 703)	Betula (n = 326)	BASE-II (n = 382)	NESDA (n = 284)	MOTAR (n = 123)
	Oxford University, UK	University of Barcelona, Spain	University of Oslo, Norway	Cambridge University, UK	Umea University, Sweden	Max Planck Institute, Germany	VU University, Netherlands	VU University, Netherlands
***Demographics***								
Age, range	60–84	63–76	19–85	18–89	25–81	24–81	18–57	19–70
Age, mean years (SD)	69.8 (5.2)	69.0 (3.2)	39.0 (15.0)	54.8 (18.4)	61.9 (13.2)	64.9 (14.3)	37.6 (10.2)	39.2 (13.5)
Sex, female, n (%)	149 (19.4 %)	82 (68.3 %)	515 (69.4 %)	358 (50.9 %)	175 (53.7 %)	145 (37.9 %)	193 (67.9 %)	64 (52.0 %)
Education, mean years (SD)	14.7 (3.4)	11.1 (4.2)	15.8 (2.5)	16.8 (3.7)	12.9 (4.1)	14.1 (2.9)	12.8 (3.2)	13.0 (3.7)

***Depression***								
Depression scale	CES-D	HDRS	BDI	HADS	CES-D	CES-D	IDS-SR	IDS-SR
Depressive symptoms, mean score (SD)	5.3 (6.2)	2.2 (2.5)	5.0 (4.5)	2.8 (2.5)	8.0 (6.5)	6.7 (6.1)	23.5 (14.2)	21.2 (21.3)
Mild-to-severe depression, n (%)	60 (7.8 %)	3 (2.5 %)	115 (15.5 %)	36 (5.1 %)	35 (10.7 %)	38 (9.9 %)	198 (69.7 %)	55 (44.7 %)
Moderate-to-severe depression, n (%)	15 (1.9 %)	0 (0 %)	21 (2.8 %)	10 (1.4 %)	12 (3.7 %)	11 (2.9 %)	143 (50 %)	50 (40.6 %)

***Brain***								
mOFC thickness, mean in mm (SD)	2.4 (1.14)	2.4 (0.11)	2.5 (0.13)	2.5 (0.12)	2.4 (0.12)	2.4 (0.10)	2.2 (0.11)	2.6 (0.15)
rACC thickness, mean in mm (SD)	2.7 (0.18)	2.8 (0.15)	2.9 (0.17)	2.8 (0.16)	2.8 (0.18)	2.6 (0.14)	2.6 (0.14)	2.9 (0.19)
Hippocampus volume, mean in cm^3^ (SD)	3.6 (0.46)	3.8(0.32)	4.1 (0.43)	4.0 (0.52)	3.8 (0.47)	3.9 (0.42)	3.9 (0.39)	4.0 (0.37)
Total GMV, mean in cm^3^ (SD)	606.3 (50.78)	585.9 (46.57)	679.0 (66.69)	651.1 (71.06)	632.2 (61.18)	602.0 (55.69)	643.2 (59.70)	616.5 (66.42)

Note: Abbreviations: SD = standard deviation, mOFC = medial orbitofrontal cortex, rACC = rostral anterior cingulate cortex, GMV = grey matter volume, CES-D = Center for Epidemiological Studies-Depression Scale, HDRS = Hamilton Depression Rating Scale, BDI = Beck Depression Inventory, HADS = Hospital Anxiety and Depression Scale, IDS-SR = Inventory of Depressive Symptomatology – Self Report.

**Fig. 1:**
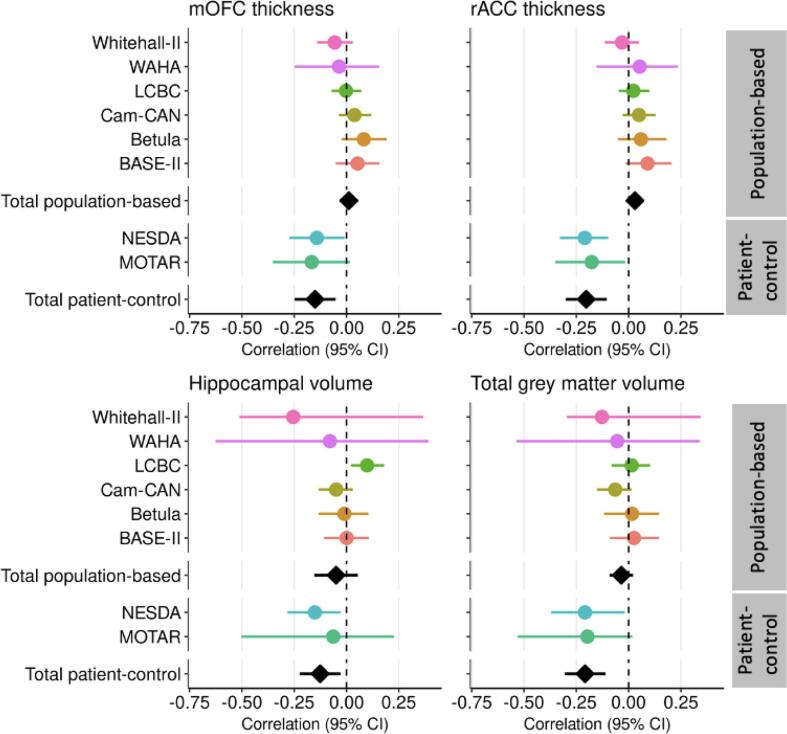
Forest plots of associations between depressive symptom scores and brain structure. Forest plots illustrating the linear associations of depressive symptom scores with thickness of medial orbitofrontal cortex (mOFC) and rostral anterior cingulate cortex (rACC), and hippocampal and total grey matter volume in the different cohorts (coloured circles), with random model pooled effect sizes (black diamonds) separately across population-based and patient-control cohorts (adjusted for age, sex, scanner and volumetric measures for intracranial volume). Horizontal lines represent 95% confidence intervals (CI).

### 3.2 Associations between depressive symptom scores and brain structure

In the pooled population-based cohorts, depressive symptom scores were not significantly associated with any brain measure of interest (Fig. 1 and Table S2, *r* = −0.05 – *r* = 0.03, *p* = 0.133 – *p* = 0.623). In the pooled patient-control cohorts, depressive symptoms were significantly negatively associated with mOFC thickness (*r* = −0.15, *95 % CI =* −0.25 to −0.05, *p* = 0.003), rACC thickness (*r* = −0.20, *95 % CI* = −0.30 to −0.10*, p* < 0.001), hippocampal volume (*r* = −0.13, *95 % CI* = −0.22 to −0.03, *p* = 0.012), and total GMV (*r* = −0.21, *95 % CI* = −0.31 to −0.11, *p* < 0.001). Results remained similar when also correcting for years of education. In meta-regressions, Freesurfer version was not associated with differences in effect size across cohorts for any of the included brain structures (Table S5).

### 3.3 Associations between dichotomous depression status and brain structure

**Fig. 2:**
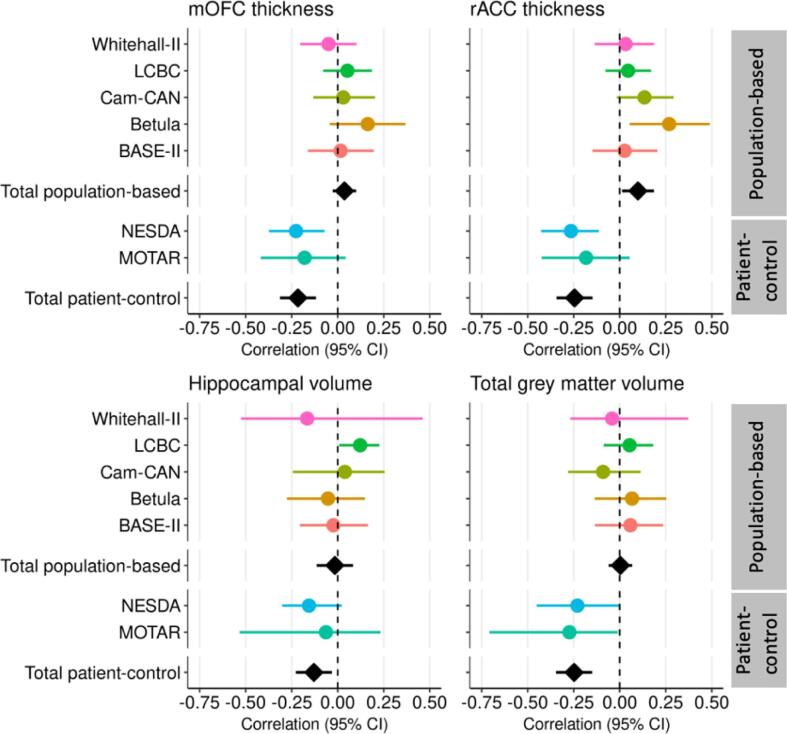
Forest plots: Associations of mild-to-severe depression vs no depression with brain structure. Forest plots illustrating the associations of mild-to-severe depression compared to no depression with thickness of medial orbitofrontal cortex (mOFC) and rostral anterior cingulate cortex (rACC), and hippocampal and total grey matter volume in the different cohorts (coloured circles), with random model pooled effect sizes (black diamonds) separately across population-based and patient-control cohorts (adjusted for age, sex, scanner and volumetric measures for intracranial volume). Horizontal lines represent 95% confidence intervals (CI).

In the population-based cohorts, there were no associations of mild-to-severe depression with any of the brain structures (Fig. 2, Table S2, *r* = −0. 016 – *r* = 0.099, *p* = 0.025 – *p* = 0.911). In the patient-control cohorts, mild-to-severe depression was negatively associated with mOFC (*r* = −0.22, *95 % CI* = −0.31 to −0.12, *p* < 0.001), rACC thickness (*r* = −0.25, *95 % CI* = −0.34 to −0.15, *p* < 0.001), hippocampal volume (*r* = −0.13, *95 % CI* = −0.23 to −0.03, *p* = 0.009), and total GMV (*r* = −0.25, *95 % CI* = −0.35 to −0.15, *p* < 0.001).

Sensitivity analyses, comparing moderate-to-severe depression cases with participants without depression (and thus excluding mildly depressed participants), still did not yield any association with brain structures in population-based cohorts (Fig. S2). In patient-control cohorts, moderate-to-severe depression was associated with low mOFC (*r* = −0.24, *95 % CI* = −0.33 to −0.14, *p* < 0.001) and rACC (*r* = −0.28, *95 % CI* = −0.40 to −0.17, *p* < 0.001) thickness, hippocampal volume (*r* = −0.15, *95 % CI* = −0.28 to −0.01, *p* = 0.037), and total GMV (*r* = −0.26, *95 % CI* = −0.36 to −0.17, *p* < 0.001).

### 3.4 Effect of age and sex

**Fig. 3:**
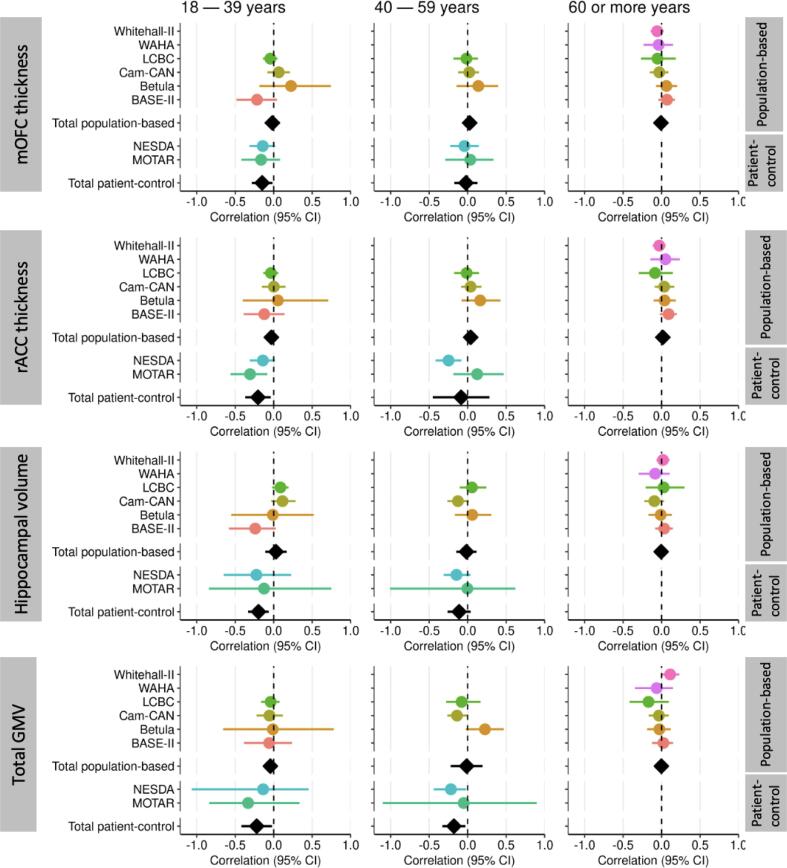
Associations of depressive symptoms with thickness of mOFC, rACC and hippocampal volume per age category. Forest plots illustrating the associations of depressive symptom scores with thickness of medial orbitofrontal cortex (mOFC) and rostral anterior cingulate cortex (rACC), and hippocampal and total grey matter volume in the different cohorts (coloured circles), with random model pooled effect sizes (black diamonds) across the age categories (18–39 years, 40–59 years, 60 or more years) (adjusted for sex, scanner and volumetric measures for intracranial volume). Horizontal lines represent 95 % confidence intervals (CI).

Across samples, thickness of the mOFC and rACC showed a linear negative association with age (Fig. S3). Hippocampal volume was relatively stable until about 60 years and then showed a decline with age. No consistent patterns were observed when plotting associations between depressive symptoms and brain structure separately for different age groups (18–39, 40–59, 60 or more years), for both population-based and patient-control cohorts (Fig. 3 and Table S3). However, no estimations could be made for late life (60 or more years) in patient-control cohorts, due to limited number of participants in this age group (Table S4). Also when splitting the oldest age group into two additional age bins of 60 to 69 years and 70 years or older, no differences were observed between age groups.

Similarly, no consistently different patterns were visible when plotting associations between depressive symptoms and brain structures separately per sex (Fig. S4 and Table S3). Meta-regressions showed that proportion of women per cohort did not influence results (Table S5).

## 4 Discussion

We investigated associations of depressive symptom severity and depression scale derived depression status, measured at the time of scan, with four brain structures, by pooling data of 3,449 participants from eight European cohorts. In clinical patient-control cohorts, depressive symptoms and depression status were significantly associated with lower mOFC, rACC thickness, hippocampal volume and total GMV, albeit effect sizes were small. In population-based cohorts, no consistent associations of the included brain regions with depressive symptoms were found, suggesting that the structural brain differences found in this study are limited to clinical samples. Associations were similar across age groups and for men and women.

Depressive symptom scores, as well as mild-to-severe depression status, were associated with lower mOFC and rACC thickness, and with lower hippocampal volume in patient-control cohorts. This may indicate a dose–response relationship and is in line with large meta-analyses relating clinical depression to lower mOFC and rACC thickness (Schmaal et al., 2017) and lower hippocampal volume (Schmaal et al., 2016). When comparing effect sizes of the current study and the meta-analyses by Schmaal et al. (Schmaal et al., 2016, Schmaal et al., 2017) by calculating Cohen’s d based on the pooled correlation coefficients of our analyses on mild-to-severe depression within the patient-control cohorts, the effect sizes of the current study were slightly larger than those observed by Schmaal et al. (Schmaal et al., 2016, Schmaal et al., 2017) (mOFC: −0.3 vs −0.13 (left and right), rACC: −0.41 vs −0.13 (left)/0.10 (right), hippocampus: −0.25 vs −0.14). More severe depressive symptoms and the presence of depression were unexpectedly also related to lower total GMV in the patient-control cohorts. This may indicate that depression could also be associated with global brain differences. Findings on associations of global brain measures and depression are inconsistent. While one meta-analysis (Arnone et al., 2016) did not find any associations with global brain measures, such as whole brain grey matter, another study (Patel et al., 2015) reported whole brain measures to predict late life depression. The association of lower total GMV with depression could also be driven by the region-specific effects that we found or potential other localized (unilateral) effects of brain regions that were not included in the current study. Previously reported results on these regions not included in our study were inconsistent and had smaller effect sizes (Arnone et al., 2016, Schmaal et al., 2016, Schmaal et al., 2017). More research is needed to confirm widespread differences of the brain in relation to depression.

In population-based cohorts, no consistent associations were found between depression and the examined brain measures. The absence of consistent associations between depression and regional measures in population-based cohorts is not in line with some other studies demonstrating associations with mild depressive symptoms (Besteher et al., 2020). However, in those studies only associations with certain brain regions such as OFC and ACC were observed, but not with the hippocampus (Webb et al., 2014, Dotson et al., 2009, Taki et al., 2005). An earlier study from one of the included samples (Whitehall-II) also found no association between depressive symptoms and global grey matter measures (Allan et al., 2016). The absence of associations in population-based cohorts could be due to the low level of depressive symptoms in these samples, with only relatively few participants having mild or moderate-to-severe depression. Between 3 % and 16 % had at least mild depressive symptoms and less than 3 % had at least moderate depressive symptoms. This is lower than the estimated prevalence of depression in the general population of 4.4 % (World Health Organization, 2017) which could partly be explained by exclusion of participants with a psychiatric history in some of the population-based cohorts. However, our findings could also suggest that the mild depressive symptoms most often observed in the general population are not related to differences in the brain structures we investigated. Depressive symptoms in the population-based samples could be more of a state measure not related to differences in brain structure but careful interpretation of the findings is warranted as levels of depression are low.

Taken together, our findings suggest that lower thickness and volumes of the investigated brain regions are only associated with clinical levels of depression and not with mild depressive symptoms. Compared to milder depressive symptoms, clinical depression is more heritable, has an earlier period of development with a peak in adolescence, is a more severe and often a chronic, recurrent condition and related to several neurobiological dysregulations (Otte et al., 2016). The observed brain differences could therefore be related to these characteristics of clinical depression. Lower thickness and volume could be a consequence of depression episodes and be related to other disturbances also seen in depression, such as inflammation (Byrne et al., 2016), lower brain-derived neurotrophic factor (BDNF) levels (Polyakova et al., 2020), or increased activity of HPA-axis related processes (Frodl and O’Keane, 2013). On the other hand, lower thickness and volume might also be related to risk factors indicating vulnerability for developing depression, such as familial risk (Zhang et al., 2020) or (early) life stress (Ho and King, 2021, Frodl et al., 2017), which might underlie both development of depression as well as the mean brain differences seen in depression. However, whether brain differences reflect vulnerability or long-term ‘scarring’ consequences cannot be concluded from our cross-sectional study. Longitudinal studies have suggested that differences might be related to depression onset or vulnerability rather than be the result of depressive episodes (Binnewies et al., 2021, Demnitz et al., 2020), although findings are inconsistent (Dohm et al., 2017).

While age was negatively associated with brain structure across studies, especially with hippocampal volume and total GMV (Fig. S3), associations of depression with brain measures were consistent across age groups. Within the oldest group (60 years or older), no consistent associations were found in the population-based cohorts, even when we split the age group in 60 to 70 years and 70 years and older. Furthermore, results were consistent for men and women, in line with lack of sex-differences previously observed in ENIGMA meta-analyses (Schmaal et al., 2016, Schmaal et al., 2017).

Some limitations of the current study should be kept in mind. In the population-based cohorts, the prevalence of depression was lower than expected based on general population prevalence, potentially due to selection bias in the studies, as history of psychiatric illness was an exclusion criterion in some of the studies. However, given the large sample size of the population-based cohorts, it is unlikely that null findings are only due to limited number of depressed cases. There may also be a sampling bias in the population-based studies as participants may not take part in research when currently not feeling well which may not be the case for participants of patient-control studies which may be included in a clinical setting. Also, depression status was based on depression scales and not clinician-rated instruments measuring clinical depression. However, validated cut-offs were used to determine thresholds for depression status. For most of the population-based cohorts, information on clinical characteristics such as use of antidepressant medication or (history of) mental disorders was limited. Thus, although their influence is likely limited, these factors could not be accounted for as potentially influencing associations in the current study. For some of the population-based cohorts, antidepressant-use was an exclusion criterion, while data on antidepressant-use was not available for the other population-based cohorts. Associations between depression and the included brain structures in the patient-control cohorts are not likely to be influenced by current antidepressant-use; in MOTAR participants with current antidepressant-use were excluded, and in NESDA we have previously shown that correcting for antidepressant-use did not change associations of depression and the included brain regions of interest (Binnewies et al., 2021). Another limitation is that the age range of the patient-control cohorts was mainly restricted to young and middle-aged adults, but older age groups were well represented in the population-based cohorts, and that only two patient-control cohorts were included. Yet, our findings can still be relevant as the cohorts were well phenotyped and clinically homogenous. Also, other factors potentially influencing both depression as well as brain measures, such as stress or lifestyle, were not taken into account.

For the current study, we restricted the number of regions of interest and only included regions that have been most strongly linked with depression (Arnone et al., 2016, Schmaal et al., 2016, Schmaal et al., 2017) as we expected rather small effect sizes based on earlier studies on (clinical) depression (Schmaal et al., 2016, Schmaal et al., 2017), limited depressive symptoms in the population-based cohorts, and relatively small samples including participants with clinical depression. Potentially, methodological differences between studies may have induced bias, such as different Freesurfer versions, scanner parameters, or depression instruments. Where possible, we have tried to account for these differences by choosing similar depression instruments, performing meta-regressions, and controlling for confounders, which all suggested that these factors did not influence our results. Another potential methodological limitation is the use of age groups to explore effects of age on associations of depression and the included brain structures. While other approaches for analysing age effects, such as meta-regression or interaction analyses might often be preferable, dividing the cohorts into age groups was the best approach for the current study due to the differences in mean age and age ranges across cohorts.

Future studies should investigate clinical as well as mild depression in broad age ranges, including adequate sample sizes of children as well as the oldest age groups, to further explore potential age effects. Future studies would benefit from including more brain regions and more detailed information on (characteristics of) potential clinical depression, such as age of onset, recurrence of potential clinical depression and antidepressant medication, when investigating population-based cohorts. Despite these limitations, the strengths of our study include the large sample size, inclusion of cohorts with different populations, access to individual level data from all samples allowing for structured and consistent data analyses across cohorts, and the inclusion of population-based as well as patient-control cohorts, allowing for investigation of associations within and across these populations.

To conclude, using pooled analyses we found that only in clinical cohorts but not in population-based cohorts, depression was modestly associated with lower thickness of mOFC and rACC, and hippocampal and total grey matter volume. These effects were also consistent over the adult lifespan and between sexes. This suggests that only in clinical populations, with more severe depressive symptoms, significant structural brain differences are detectable, whereas this is not the case for the more prevalent milder depressive symptoms seen in population-based samples. Differences in brain structure could be related to vulnerability or characteristics of clinical depression such as chronicity or severity, or be a long-term consequence of clinical depression, and more research is needed to gain further insight into the association between clinical depression and brain structure.

## CRediT authorship contribution statement

**Julia Binnewies:** Writing – original draft, Methodology, Formal analysis, Visualization, Conceptualization. **Laura Nawijn:** Writing – review & editing, Methodology, Supervision, Conceptualization. **Andreas M. Brandmaier:** Methodology, Writing – original draft. **William F.C. Baaré:** Writing – original draft. **David Bartrés-Faz:** Writing – original draft, Investigation, Funding acquisition. **Christian A. Drevon:** Writing – review & editing. **Sandra Düzel:** Data curation. **Anders M. Fjell:** Writing – review & editing, Investigation, Funding acquisition. **Laura K.M. Han:** Data curation, Writing – review & editing. **Ethan Knights:** Writing – review & editing, Data curation. **Ulman Lindenberger:** Investigation, Funding acquisition. **Yuri Milaneschi:** Writing – review & editing, Methodology. **Athanasia M. Mowinckel:** Software, Data curation. **Lars Nyberg:** Writing – review & editing, Investigation, Funding acquisition. **Anna Plachti:** Writing – review & editing. **Kathrine Skak Madsen:** Writing – review & editing. **Cristina Solé-Padullés:** Writing – review & editing, Data curation. **Sana Suri:** Writing – review & editing. **Kristine B. Walhovd:** Writing – review & editing, Funding acquisition. **Enikő Zsoldos:** Writing – review & editing, Funding acquisition. **Klaus P. Ebmeier:** Writing – review & editing, Investigation, Funding acquisition. **Brenda W.J.H. Penninx:** Writing – review & editing, Methodology, Funding acquisition, Supervision, Conceptualization.

## Declaration of Competing Interest

The authors declare that they have no known competing financial interests or personal relationships that could have appeared to influence the work reported in this paper.

## Data Availability

Data will be made available on request.
