# Supplementary material for: Associations of depression and regional brain structure across the adult lifespan: Pooled analyses of six population-based and two clinical cohort studies in the European Lifebrain consortium
Source: Neuroimage Clin. 2022 Sep 5;36:103180. doi: 10.1016/j.nicl.2022.103180 (PMC9467888; doi:10.1016/j.nicl.2022.103180)
Supplement: Supplementary data 1 [file mmc1.docx]

**Supplementary material for *Associations of depression and regional brain structure across the adult lifespan: Pooled analyses of 6 population-based and 2 clinical cohort studies in the European Lifebrain consortium***

Julia Binnewies, Laura Nawijn, Andreas M Brandmaier, William FC Baaré, David Bartrés-Faz, Christian A Drevon, Sandra Düzel, Anders M Fjell, Laura KM Han, Ethan Knights, Ulman Lindenberger, Yuri Milaneschi, Athanasia M Mowinckel, Lars Nyberg, Anna Plachti, Kathrine Skak Madsen, Cristina Solé-Padullés, Sana Suri, Kristine B Walhovd, Enikő Zsoldos, Klaus P Ebmeier, Brenda WJH Penninx

**Table S1. Depression scales and caseness thresholds by cohort**

|  | **Population-based cohorts** | | | | | | **Patient-control cohorts** | |  |
| --- | --- | --- | --- | --- | --- | --- | --- | --- | --- |
|  | Whitehall-II | WAHA | LCBC | Cam-CAN | Betula | BASE-II | NESDA | MOTAR | |
|  | Oxford University, UK | University of Barcelona, Spain | University of Oslo, Norway | Cambridge University, UK | Umea University, Sweden | Max Planck Institute, Germany | VU University, Netherlands | VU University, Netherlands | |
| **Depression** |  |  |  |  |  |  |  |  | |
| ***Depression scale*** | 20-item Center for Epidemiological Studies-Depression Scale (CES-D) | Hamilton Depression Rating Scale (HDRS) | Beck Depression Inventory (BDI) | Hospital Anxiety and Depression Scale (HADS) | 20-item Center for Epidemiological Studies-Depression Scale (CES-D) | 20-item Center for Epidemiological Studies-Depression Scale (CES-D) | 30-item Inventory of Depressive Symptomatology - Self Report (IDS-SR) | 30-item Inventory of Depressive Symptomatology - Self Report (IDS-SR) | |
| ***Thresholds*** |  |  |  |  |  |  |  |  | |
| Mild-to-severe | ≥16 | ≥8 | ≥10 | ≥8 | ≥16 | ≥16 | ≥14 | ≥14 | |
| Moderate-to-severe | ≥23 | ≥17 | ≥17 | ≥11 | ≥23 | ≥23 | ≥26 | ≥26 | |
| **MRI** |  |  |  |  |  |  |  |  | |
| Scanner | Verio Siemens; Prisma Siemens | Tim Trio Siemens | Avanto Siemens; Skyra Siemens; Prisma Siemens | Tim Trio Siemens | Discovery GE | Tim Trio Siemens | Philips | Philips | |
| Tesla | 3.0 | 3.0 | 1.5; 3.0; 3.0 | 3.0 | 3.0 | 3.0 | 3.0 | 3.0 | |
| Freesurfer version | 5.3 | 6.0 | 6.0 | 6.0 | 6.0 | 7.0 | 6.0 | 6.0 | |


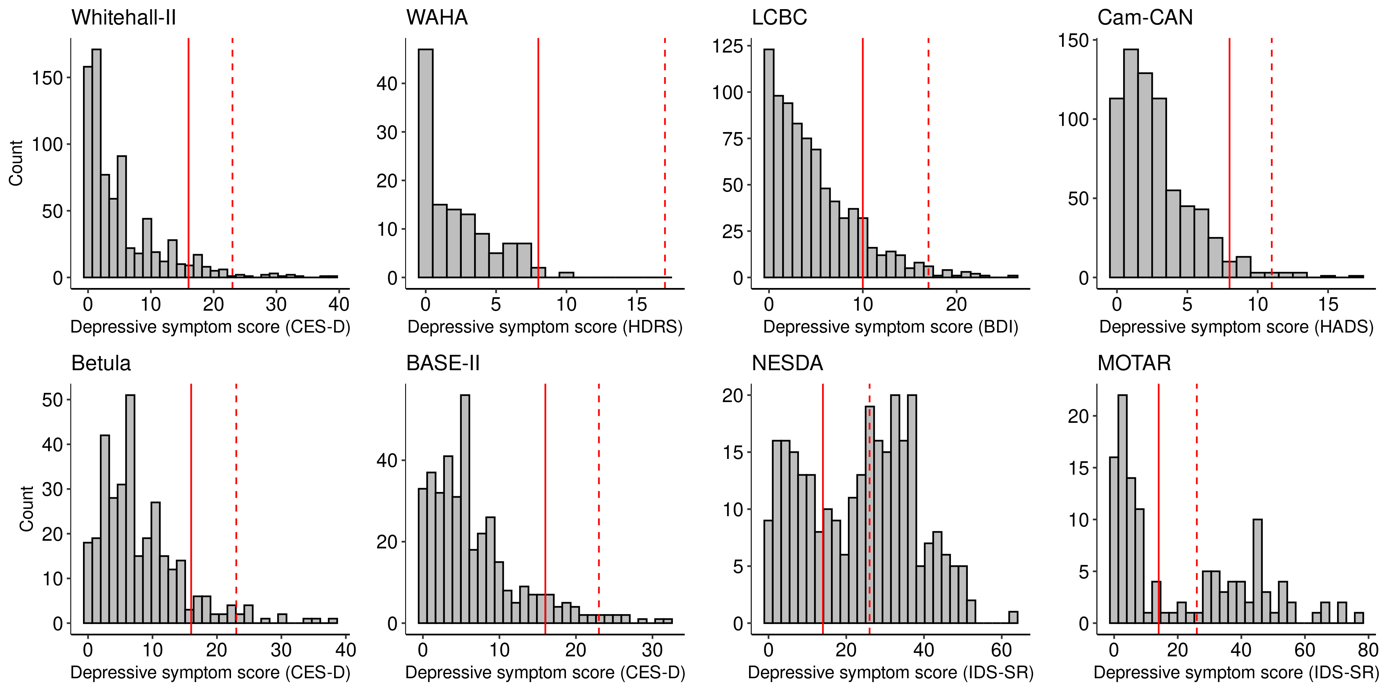


**Figure S1. Histograms showing the distribution of depressive symptoms per cohort.** Histograms showing the distribution of depressive symptom score of the respective depression instrument (20-item Center for Epidemiological Studies-Depression Scale (CES-D), Hamilton Depression Rating Scale (HDRS), Beck Depression Inventory (BDI), Hospital Anxiety and Depression Scale (HADS), 30-item Inventory of Depressive Symptomatology - Self Report (IDS-SR)) for each cohort. Solid red line indicating threshold for mild-to-severe depression, dashed red line indicating threshold for moderate-to-severe depression.

**Table S2. Associations of depressive symptoms scores and presence of mild-to-severe depression with brain structure**

|  | **mOFC thickness** | **rACC thickness** | **Hippocampal volume** | **Total grey matter volume** |
| --- | --- | --- | --- | --- |
|  | r (95% CI), p | r (95% CI), p | r (95% CI), p | r (95% CI), p |
| **Depressive symptoms** |  |  |  |  |
| LCBC | -0.002 (-0.07 to 0.07), 0.948 | 0.022 (-0.05 to 0.10), 0.581 | 0.098 (0.02 to 0.18), 0.014 | 0.014 (-0.08 to 0.10), 0.755 |
| Whitehall | -0.056 (-0.14 to 0.03), 0.171 | -0.032 (-0.11 to 0.05), 0.384 | -0.255 (-0.51 to 0.37), 0.055 | -0.128 (-0.29 to 0.34), 0.141 |
| CamCAN | 0.038 (-0.04 to 0.12), 0.349 | 0.050 (-0.03 to 0.13), 0.226 | -0.049 (-0.13 to 0.03), 0.279 | -0.064 (-0.15 to 0.01), 0.140 |
| Betula | 0.083 (-0.02 to 0.19), 0.142 | 0.059 (-0.05 to 0.18), 0.363 | -0.010 (-0.13 to 0.10), 0.877 | 0.016 (-0.12 to 0.14), 0.825 |
| WAHA | -0.036 (-0.25 to 0.16), 0.709 | 0.053 (-0.15 to 0.24), 0.559 | -0.079 (-0.63 to 0.39), 0.550 | -0.055 (-0.54 to 0.34), 0.746 |
| BASE | 0.053 (-0.05 to 0.16), 0.331 | 0.090 (-0.01 to 0.21), 0.104 | 0.001 (-0.11 to 0.11), 0.934 | 0.026 (-0.09 to 0.15), 0.667 |
| *Total population-based* | *0.011 (-0.03 to 0.06), 0.623* | *0.030 (-0.01 to 0.07), 0.133* | *-0.052 (-0.15 to 0.05), 0.347* | *-0.035 (-0.09 to 0.02), 0.223* |
| NESDA | -0.142 (-0.27 to -0.01), 0.029 | -0.210 (-0.33 to -0.110), 0.001 | -0.151 (-0.28 to -0.03), 0.018 | -0.209 (-0.37 to -0.02), 0.004 |
| MOTAR | -0.166 (-0.35 to 0.02), 0.070 | -0.177 (-0.35 to 0.21), 0.048 | -0.063 (-0.50 to 0.23), 0.692 | -0.197 (-0.53 to 0.02), 0.151 |
| *Total patient-control* | *-0.150 (-0.25 to -0.05),* ***0.003*** | *-0.203 (-0.30 to -0.10),* ***<0.001*** | *-0.126 (-0.22 to -0.03),* ***0.012*** | *-0.208 (-0.31 to -0.11),* ***<0.001*** |
| **Depression caseness** |  |  |  |  |
| LCBC | 0.053 (-0.08 to 0.19), 0.378 | 0.045 (-0.08 to 0.17), 0.499 | 0.122 (0.01 to 0.23), 0.029 | 0.053 (-0.09 to 0.18), 0.432 |
| Whitehall | -0.050 (-0.20 to 0.10), 0.549 | 0.032 (-0.14 to 0.19), 0.704 | -0.166 (-0.53 to 0.46), 0.399 | -0.042 (-0.27 to 0.37), 0.721 |
| CamCAN | 0.030 (-0.13 to 0.20), 0.682 | 0.134 (-0.02 to 0.29), 0.114 | 0.039 (-0.24 to 0.25), 0.833 | -0.091 (-0.28 to 0.11), 0.405 |
| Betula | 0.163 (-0.04 to 0.37), 0.117 | 0.269 (0.05 to 0.49), 0.026 | -0.053 (-0.28 to 0.15), 0.637 | 0.067 (-0.14 to 0.25), 0.472 |
| BASE | 0.016 (-0.16 to 0.20), 0.846 | 0.027 (-0.15 to 0.20), 0.781 | -0.024 (-0.21 to 0.16), 0.774 | 0.058 (-0.13 to 0.24), 0.476 |
| *Total population-based* | *0.037 (-0.03 to 0.10), 0.260* | *0.099 (0.01 to 0.19), 0.025* | *-0.016 (-0.11 to 0.08), 0.757* | *0.004, (-0.06 to 0.07), 0.911* |
| NESDA | -0.227 (-0.37 to -0.07), 0.003 | -0.265 (-0.43 to -0.11), 0.001 | -0.156 (-0.30 to 0.02), 0.034 | -0.230 (-0.45 to 0.01), 0.045 |
| MOTAR | -0.180 (-0.42 to 0.04), 0.168 | -0.183 (-0.42 to 0.05), 0.137 | -0.064 (-0.53 to 0.23), 0.694 | -0.273 (-0.71 to -0.01), 0.127 |
| *Total patient-control* | *-0.216 (-0.31 to -0.12),* ***<0.001*** | *-0.246 (-0.34 to -0.15),* ***<0.001*** | *-0.129 (-0.23 to -0.03),* ***0.009*** | *-0.248 (-0.35 to -0.15),* ***<0.001*** |

Note: Abbreviations: mOFC=medial orbitofrontal cortex, rACC=rostral anterior cingulate cortex, GMV=grey matter volume, r=correlation coefficient, CI=confidence interval. Per site correlations adjusted for age, sex, scanner and volumetric measures for intracranial volume, pooled separately across population-based and patient-control cohorts. Significant pooled associations after correction for multiple testing are indicated in bold.


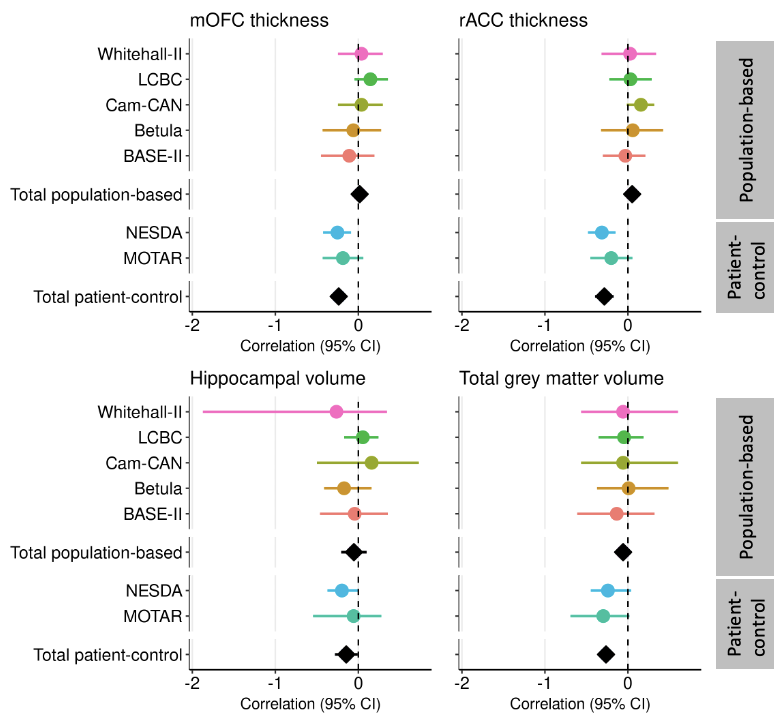


**Figure S2. Associations of moderate-to-severe depression vs no depression with thickness of mOFC, rACC and hippocampal volume.** Forest plots illustrating the associations of moderate-to-severe depression compared to no depression with thickness of medial orbitofrontal cortex (mOFC) and rostral anterior cingulate cortex (rACC), and hippocampal and total grey matter volume in the different cohorts (coloured circles), with random model pooled effect sizes (black diamonds) separately across population-based and patient-control cohorts (adjusted for age, sex, scanner and volumetric measures for intracranial volume). Horizontal lines represent 95% confidence intervals (CI).


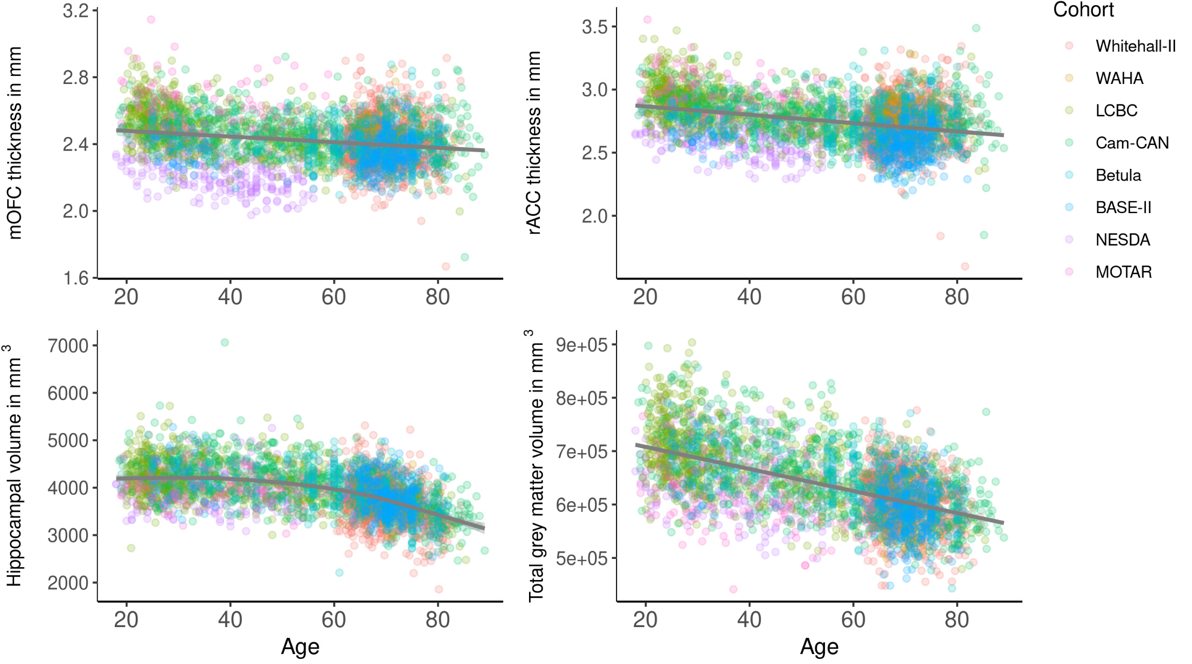


**Figure S3. Associations of thickness of mOFC, rACC, hippocampal volume, and total GMV with age across cohorts.**

**Table S3. Associations of depressive symptoms with thickness of mOFC, rACC and hippocampal volume per age category and per sex**

|  | **mOFC thickness** | **rACC thickness** | **Hippocampal volume** | **Total grey matter volume** |
| --- | --- | --- | --- | --- |
|  | r (95% CI) | r (95% CI) | r (95% CI) | r (95% CI) |
| **Age** |  |  |  |  |
| *18-40 years* |  |  |  |  |
| Total population-based | -0.017 (-0.11 to 0.08) | -0.030 (-0.10 to 0.04) | 0.030 (-0.11 to 0.17) | -0.043 (-0.12 to 0.03) |
| Total patient-control | -0.15 (-0.28 to -0.02) | -0.204 (-0.37 to -0.04) | -0.1978 (-0.33 to -0.06) | -0.220 (-0.42 to -0.02) |
| *40-60 years* |  |  |  |  |
| Total population-based | 0.026 (-0.06 to 0.12) | 0.039 (-0.05 to 0.13) | -0.016 (-0.15 to 0.12) | -0.013 **(**-0.22 to 0.19**)** |
| Total patient-control | -0.022 (-0.17 to 0.13) | -0.085 (-0.45 to 0.28) | -0.111 (-0.26 to 0.04) | -0.178 **(**-0.33 to -0.03**)** |
| *60 or more years* |  |  |  |  |
| Total population-based | -0.008 (-0.06 to 0.05) | 0.016 (-0.04 to 0.07) | -0.005 (-0.05 to 0.04) | -0.003 (-0.08 to 0.07) |
| Total patient-control | - | - | - | - |
| **Sex** |  |  |  |  |
| *Women* |  |  |  |  |
| Total population-based | 0.008 (-0.047 to 0.06) | 0.046 (-0.01 to 0.10) | 0.027 (-0.04 to 0.09) | -0.023 (-0.08 to 0.03) |
| Total patient-control | -0.176 (-0.30 to -0.05) | -0.162 (-0.29 to -0.04) | -0.173 (-0.30 to -0.05) | -0.1156 (-0.24 to 0.01) |
| *Men* |  |  |  |  |
| Total population-based | 0.052 (-0.01 to 0.11) | 0.063 (-0.01 to 0.13) | -0.027 (-0.09 to 0.04) | -0.025 (-0.09 to 0.04) |
| Total patient-control | -0.098 (-0.28 to 0.08) | -0.261 (-0.42 to -0.10) | -0.058 (-0.22 to 0.11) | -0.262 (-0.42 to -0.10) |

Note: Abbreviations: mOFC=medial orbitofrontal cortex, rACC=rostral anterior cingulate cortex, GMV=grey matter volume, r=correlation coefficient, CI=confidence interval. Per site correlations per age bin adjusted for sex, scanner and volumetric measures for intracranial volume, and per site correlations per sex adjusted for age, scanner and volumetric measures for intracranial volume, pooled separately across population-based and patient-control cohorts.

**Table S4.** **Sample sizes per age category per cohort**


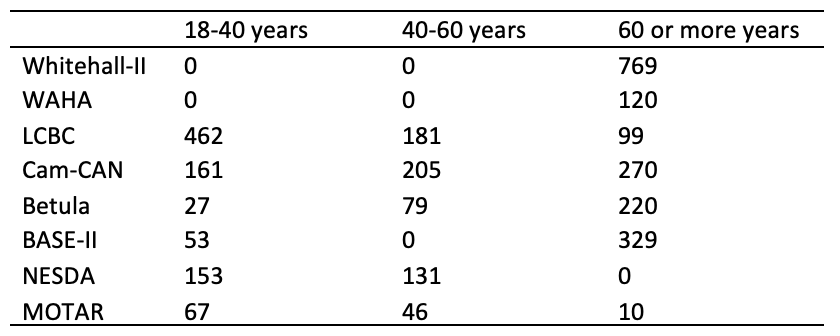


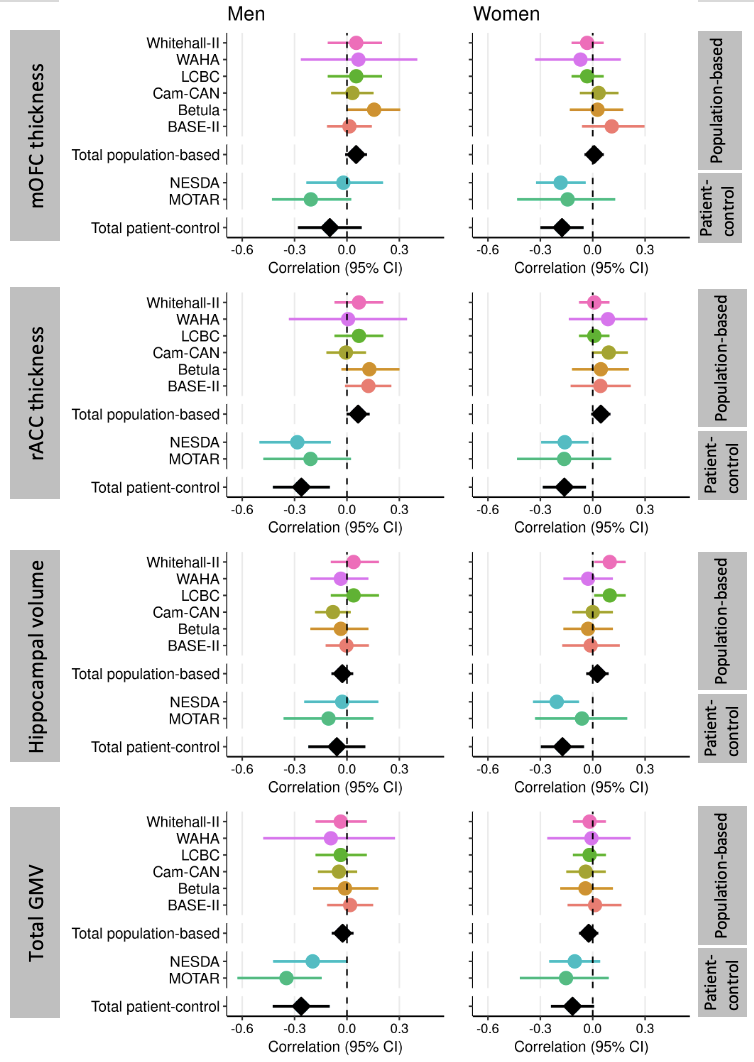


**Figure S4. Associations of depressive symptoms with thickness of mOFC, rACC and hippocampal volume separate for women and men.** Forest plots illustrating the associations of depressive symptom scores with thickness of medial orbitofrontal cortex (mOFC) and rostral anterior cingulate cortex (rACC), and hippocampal and total grey matter volume in the different cohorts (coloured circles), with random model pooled effect sizes (black diamonds) across sexes (adjusted for age, scanner and volumetric measures for intracranial volume). Horizontal lines represent 95% confidence intervals (CI).

**Table S5. Meta-regressions on effect of Freesurfer version and proportion of women per site on associations of depressive symptoms with thickness of mOFC, rACC and hippocampal volume**

|  | **mOFC thickness** | **rACC thickness** | **Hippocampal volume** | **Total grey matter volume** |
| --- | --- | --- | --- | --- |
|  | r (95% CI), p | r (95% CI), p | r (95% CI), p | r (95% CI), p |
| Freesurfer version | 0.053 (-0.05 to 0.15), 0.29 | 0.058 (-0.09 to 0.20), 0.80 | 0.136 (0.00 to 0.27), 0.05 | 0.08 (-0.03 to 0.19), 0.17 |
| Percentage women | -0.045 (-0.41 to 0.32), 0.81 | -0.107 (-0.58 to 0.37), 0.66 | 0.38 (-0.04 to 0.81), 0.08 | 0.037 (-0.37 to 0.45), 0.86 |

Note: Abbreviations: mOFC=medial orbitofrontal cortex, rACC=rostral anterior cingulate cortex, GMV=grey matter volume, r=correlation coefficient, CI=confidence interval. Per site correlations adjusted for age, sex, scanner and volumetric measures for intracranial volume, pooled across all cohorts. Meta-regressions performed on the used Freesurfer version (version 5.3, 6.0 or 7.0) and percentage women per cohort.

**Additional information per cohort**

## **Population-based cohorts**

The studies included in the pooled analyses are derived from major European neuroimaging studies included in the Lifebrain consortium (Walhovd et al., 2018). Descriptions of each cohort are provided below.

### **BASE-II**

*Sample*

Berlin Study of Aging‐II, Charité University, Max Planck Institute, Berlin, Germany.

*Population*

Community‐dwelling older adults recruited from the greater Berlin metropolitan area.

*General description & procedures*

Participants were community‐dwelling older adults recruited from the greater Berlin metropolitan area through advertisements in newspapers and public areas. Participants were recruited within the Berlin Aging Study II (BASE‐II) (for cohort characteristics and additional details, see Bertram et al., 2014; Gerstorf et al., 2016). The baseline sample comprised 2200 participants. After completion of BASE‐II baseline measures, eligible participants were invited to take part in one MRI session within a time window of 2–4 weeks after baseline testing, consisting of 341 older adults aged 61–82 years (mean age 70.1, SD = 3.89; 131 female) and 103 younger adults (mean age 31.4, SD = 3.7; 39 female). The different elements of the study were approved by the ethics committees of the Max Planck Institute for Human Development, the Charité University ethics committee and by the ethics committees of DGPs. Participants signed written informed consent and received monetary compensation for their participation in BASE‐II and the MRI study. All experiments were performed in accordance with relevant guidelines and regulations.

*Inclusion- & exclusion criteria*

Inclusion criteria for taking part in this study were age between 20 and 35 or 60 and 80 years, apparently healthy. Exclusion criteria were untreated diabetes and hypertension; prior stroke, head injuries or brain surgery; psychiatric illness; major depression; dementia with a score < 24 on the Mini-Mental State Examination. To that end, none of the participants took medication that might affect memory function or had a history of head injuries, medical (e.g., heart attack), neurological (e.g., epilepsy), or psychiatric disorders (e.g., depression). All participants reported normal or corrected to normal vision, were right-handed, and scored over 27 on the Mini-Mental Status Examination.

*Depressive symptoms*

Depressive symptoms were assessed using the 20-item Center for Epidemiological Studies-Depression Scale (CES-D).

*MRI measures*

Imaging data were acquired using a Tim Trio Siemens 3.0 Tesla scanner with the following parameters: TR: 2,500 ms, TE: 4.77 ms, TI: 1,100 ms, flip angle: 7°, slice thickness: 1.0 mm, FoV 256 × 256 mm, 176 slices.

*Key references*

- Bertram, L., Böckenhoff, A., Demuth, I., Düzel, S., Eckardt, R., Li, S.‐C. C., … Steinhagen‐Thiessen, E. (2014). Cohort profile: The Berlin Aging Study II (BASE‐II). International journal of epidemiology,43(3), 703–12. doi:10.1093/ije/dyt018
- Gerstorf, D., Bertram, L., Lindenberger, U., Pawelec, G., Demuth, I., Steinhagen‐Thiessen, E., & Wagner, G. G. (2016). Editorial. Gerontology,62(3), 311–5. doi:10.1159/000441495

### **Betula**

*Sample source*

The Betula longitudinal study on aging, memory and dementia, University of Umeå, Sweden.

*Population*

Population‐based, healthy middle‐aged and older adults.

*General description & procedures*

Population‐based sampling was used for recruitment, detailed recruitment procedures are found in Nilsson et al., 1997. Participation in the neuroimaging study was offered to all participants who had remained in the study and completed cognitive testing at the 5^th^ Betula test wave in 2008‐2009. A subset of 376 participants from the longitudinal Betula study (Nilsson et al., 1997) underwent structural and functional MRI in 2009‐2010 and 232 returned for a follow‐up scan in 2013‐2014. The parent samples from which the scanned participants were derived from were originally recruited to the study in 1988, 1993, and 2008 respectively. The study was approved by the relevant ethical review board.

*Inclusion- & exclusion criteria*

Exclusion criteria were severe visual or auditory handicaps, intellectual or developmental disabilities, suspected dementia, having a mother tongue other than Swedish, MRI contraindications, neurological disorders, or visual/motor deficits that could interfere with fMRI data collection, MMSE <24, brain or head surgery, and substantial brain anatomical deviations. Eight participants were excluded completely post scanning due to discovered neurological conditions (Schizophrenia, Multiple Sclerosis, Parkinson’s Disease, Hydrocephalus, Alcoholism, and dementia), and an additional two participants were excluded due to MMSE scores below 24. In addition, for 29 participants MRI data only was excluded due to anatomical deviations (subdural hematoma, localized loss of brain tissue, subcortical atrophy, and previous brain or head surgery (n=2)), movement artifacts (n=21), or FreeSurfer processing failures (n=3). Three individuals had missing T1 images due to incomplete acquisition. Thus, the final data set comprised MRI volumetric data for 334 participants.

*Depressive symptoms*

Depressive symptoms were assessed using the 20-item Center for Epidemiological Studies-Depression Scale (CES-D).

*MRI measures*

Imaging data was acquired using a Discovery GE 3.0 Tesla scanner with the following parameters: TR: 8.19 ms, TE: 3.2 ms, TI: 450 ms, flip angle: 12°, slice thickness: 1 mm, FOV 250 × 250 mm, 180 slices.

*Key references*

- Nilsson, L.‐G., Bäckman, L., Erngrund, K., Nyberg, L., Adolfsson, R., Bucht, G., Karlsson, S., Widing, M., Winblad, B., 1997. The Betula prospective cohort study: Memory, health, and aging. Aging, Neuropsychol. Cogn. 4, 1–32. doi:10.1080/13825589708256633

### **Cam-CAN**

*Sample source*

The Cambridge Centre for Ageing and Neuroscience (Cam‐CAN) study, Cambridge University, United Kingdom.

*Population*

Population‐based, adult lifespan (18 years and up), cognitively healthy adults.

*General description & procedures*

A population‐based cohort of 3000 adults aged 18 was recruited to Stage 1 of the project. Invitation letters were based on the patient lists of general practitioners within the Cambridge City area. At stage 1 participants completed an interview including health and lifestyle questions, a core cognitive assessment, and a self‐completed questionnaire of lifetime experiences and physical activity. Of those interviewed, ~700 participants aged 18‐87 (100 per age decile) continued to Stage 2 where they undergo cognitive testing and provide measures of brain structure and function. The study is conducted in compliance with the Helsinki Declaration, and has been approved by the local ethics committee, Cambridgeshire 2 Research Ethics Committee (reference: 10/H0308/50).

*Inclusion- & exclusion criteria*

General exclusion criteria: Term‐time residents of colleges and universities, and participants whose Primary Care Physician feel are inappropriate to include. Exclusion criteria: No cognitive impairment (MMSE < 25, memory defect, consent difficulties), communication difficulties (hearing problems [35db at 1000 Hz], insufficient English language, vision difficulties), medical problems by self‐report of diagnosis (dementia diagnosis /Alzheimer’s Disease, Parkinson’s Disease, Motor Neurone disease, Multiple sclerosis, cancer, stroke, encephalitis, meningitis, epilepsy, head injury with serious results [coma, unconscious for >2 hours, skull fracture], recently diagnosed or uncontrolled high blood pressure, possible pregnancy, current psychiatric conditions [bipolar disorder, schizophrenia, psychosis]), mobility problems (restricted mobility which could prevent further participation, inability to walk 10 metres), MRI/ MEG safety and comfort exclusions.

*Depressive symptoms*

Depressive symptoms were assessed using the Hospital Anxiety and Depression Scale (HADS).

*MRI measures*

Imaging data was acquired using a Tim Trio Siemens 3.0 Tesla scanner with the following parameters: TR: 2,250 ms, TE: 2.98 ms, TI: 900 ms, flip angle: 9°, slice thickness 1 mm, FOV 256 × 240 mm, 192 slices.

*Education*

Education was scored in categories: 1. College or university degree or higher/ 2. A levels/AS levels or equivalent/ 3. O levels/GCSEs or equivalent/ 4. CSEs or equivalent/ 5. NVQ or HND or HNC or equivalent/ 6. Other professional qualifications e.g.: nursing, teaching/ 0. None of the above 8. No answer. According to Nyberg et al (2021) we converted these to years of education by using the following conversion: {1: 16, 2: 13, 3: 11, 4: 11, 5: 13, 6: 16, 0: 7, 8: NaN}. This number was then compared to the reported age at completed continuous full-time education (subtracting school starting age(=4)), prompted by the question: “At what age did you complete your continuous full-time education?” The highest number of the two was chosen as years of education.

*Key references*

- Shafto et al. The Cambridge Centre for Ageing and Neuroscience (Cam‐CAN) study protocol: a cross‐sectional, lifespan, multidisciplinary examination of healthy cognitive ageing. BMC Neurology, 2014, 14:204. doi: 10.1186/s12883‐014‐0204‐1

Taylor, J. R., Williams, N., Cusack, R., Auer, T., Shafto, M. A., Dixon, M., ... & Henson, R. N. (2017). The Cambridge Centre for Ageing and Neuroscience (Cam-CAN) data repository: Structural and functional MRI, MEG, and cognitive data from a cross-sectional adult lifespan sample. Neuroimage, 144, 262-269.

- Nyberg, L., Magnussen, F., Lundquist, A., Baaré, W., Bartrés-Faz, D., Bertram, L., … & Fjell, A. M. (2021). Educational attainment does not influence brain aging. *Proceedings of the National Academy of Sciences*, *118*(18).

### **LCBC**

*Sample source*

Center for Lifespan Changes in Brain and Cognition, University of Oslo, Norway.

*Population*

Majority: normal, cognitively healthy participants across the lifespan.

Minority: cognitively healthy patients from gynecological (genital prolapse), urological (benign prostate hyperplasia, prostate cancer, or bladder tumor/cancer) or orthopedic (knee or hip replacement) surgery in spinal anesthesia, turning 65 years or older the year of inclusion.

*General description & procedures*

Cognitively normal participants were drawn from studies coordinated by the Research Group for Lifespan Changes in Brain and Cognition (LCBC www.oslobrains.no), approved by a Norwegian Regional Committee for Medical and Health Research Ethics. Written informed consent was obtained from all participants. Participants were recruited through newspaper and web page advertisements.

*Inclusion- & exclusion criteria*

Adult participants were screened using a standardized health interview prior to inclusion in the study. Participants with a history of self‐ or parent‐reported neurological or psychiatric conditions, including clinically significant stroke, serious head injury, untreated hypertension, diabetes, and use of psychoactive drugs within the last two years, were excluded. Further, participants reporting worries concerning their cognitive status, including memory function, were excluded. All participants 40‐80 years of age were required to score >26 and participants > 80 years > 25 on the Mini Mental State Examination according to population norms. From the sub‐population of elective surgery patients, dementia, previous stroke with sequela, Parkinson's disease, and other neurodegenerative diseases likely to affect cognitive function were initial exclusion criteria. From this pool of participants, we further selected only cognitively healthy participants based on clinical examinations at Department of Geriatric Medicine at Oslo University Hospital. For each participant, the first measurement point with depression and MRI was included in the current study.

*Depressive symptoms*

Depressive symptoms were assessed using the 20-item Center for Epidemiological Studies-Depression Scale (CES-D).

*MRI measures*

Imaging data was acquired using two Avanto Siemens 1.5 Tesla scanners (TR: 2,400 ms, TE: 3.61 ms, TI: 1,000 ms, flip angle: 8°, slice thickness: 1.2 mm, FoV: 240 × 240 m, 160 slices, in-plane acceleration = 2; TR: 2,400 ms, TE = 3.79 ms, TI = 1,000 ms, flip angle = 8, slice thickness: 1.2 mm, FoV: 240 × 240 mm, 160 slices), a Skyra Siemens 3.0 Tesla scanner (TR: 2,300 ms, TE: 2.98 ms, TI: 850 ms, flip angle: 8°, slice thickness: 1 mm, FoV: 256 × 256 mm, 176 slices) and a Prisma Siemens 3.0 Tesla scanner (TR: 2,400 ms, TE: 2.22 ms, TI: 1,000 ms, flip angle: 8°, slice thickness: 0.8 mm, FoV: 240 × 256 mm, 208 slices, in-plane acceleration = 2).

*Key references*

- Walhovd, K. B., Krogsrud, S. K., Amlien, I. K., Bartsch, H., Bjørnerud, A., Due-Tønnessen, P., ... & Fjell, A. M. (2016). Neurodevelopmental origins of lifespan changes in brain and cognition. Proceedings of the National Academy of Sciences, 113(33), 9357-9362.
- Fjell AM, Idland AV, Sala‐Llonch R, Watne LO, Borza T, Brækhus A, Lona T, Zeterberg H, Blennow K, Wyller TB, Walhovd KB. Neuroinflammation and Tau interact with amyloid in predicting sleep problems in aging independently of atrophy. Cerebral Cortex, 2018, 28, 2775‐2785.

### **WAHA**

*Sample source*

Walnuts and Healthy Aging Study, Hospital Clínic, Barcelona, Spain.

*Population*

Healthy elderly adults, cognitively normal.

*General description & procedures*

Participants were healthy elderly men and women with normal cognitive and visual

function at the time of recruitment. Participants were included as part of a randomized controlled trial investigating effects of a nutritional intervention with walnuts on normal brain aging. Only baseline data was used for the current analyses. Eligible participants were recruited via mailing study brochures (LLU) or through the non‐profit organization Institute of Aging (BCN), advertisements in the study centers, and word of mouth. Interested individuals attended an informational group meeting, completed a short medical questionnaire and signed the informed consent. All gave informed consent, in accordance with the Declaration of Helsinki (1964, last revision 2013). All study procedures were approved by the local Institutional Review Board). Recruitment and selection of participants took place between May 2012 and May 2014; the trial ended May 31, 2016.

*Inclusion- & exclusion criteria*

Inclusion criteria were age between 63 and 79 years, apparently healthy, and equally willing to be in either of the two groups. Exclusion criteria included inability to undergo neuropsychological testing; morbid obesity (BMI ≥ 40 kg/m2); uncontrolled diabetes (HbA1c > 8%); uncontrolled hypertension (on‐treatment blood pressure ≥ 150/100 mmHg); prior stroke, significant head trauma or brain surgery; relevant psychiatric illness; cognitive deterioration or dementia with a score < 24 on the Mini‐Mental State Examination; other neurodegenerative disorders like Parkinson’s disease; advanced AMD or eye‐related conditions precluding ophthalmological evaluation; prior chemotherapy; chronic illness with projected shortened lifespan; allergy to walnuts; customary use of fish oil and/or tree nuts (> 2 servings/week) and/or other relevant sources of ALA, such as flaxseed oil or soy lecithin.

*Depressive symptoms*

Depressive symptoms were assessed using the Hamilton Depression Rating Scale (HDRS).

*MRI measures*

Imaging data was acquired using a Tim Trio Siemens 3.0 Tesla scanner with the following parameters: TR: 2,300 ms, TE: 2.98, TI: 900 ms, slice thickness 1 mm, flip angle: 9°, FoV 256 × 256 mm, 240 slices.

*Key references*

- Rajaram S, Valls‐Pedret C, Cofán M, Sabaté J, Serra‐Mir M, Pérez‐Heras AM, Arechiga A, Casaroli‐Marano RP, Alforja S, Sala‐Vila A, Doménech M, Roth I, Freitas‐Simoes TM, Calvo C, López‐Illamola A, Haddad E, Bitok E, Kazzi N, Huey L, Fan J, Ros E. The Walnuts and Healthy Aging Study (WAHA): Protocol for a Nutritional Intervention Trial with Walnuts on Brain Aging. Front Aging Neurosci. 2017 Jan 10;8:333.

### **Whitehall-II**

*Sample source*

The Whitehall II imaging sub‐study, Oxford University, Oxford, United Kingdom.

*Population*

Population‐representative older adults (60‐85 years).

*General description & procedures*

The Whitehall II study, starting in 1985, includes 10.308 British civil servants followed over time, which allows exploring factors hypothesized to affect brain health and cognitive aging. At study start, phase 1 (1985-1988), the population representative cohort was in the age range 35-55 years. MRI was done in Phase 11 (2012-2013) of this study, at which time the total number participants was 6035, and the age range was 60-85 years. A random sample willing and able to give informed consent to participants in the imaging sub‐study of Whitehall II was included. Ethical approval was granted generically for the “Protocol for noninvasive magnetic resonance investigations in healthy volunteers” (MSD/IDREC/2010/P17.2) by the University of Oxford Central University/ Medical Science Division Interdisciplinary Research Ethics Committee (CUREC/MSD‐IDREC), who also approved the specific protocol: “Predicting MRI abnormalities with longitudinal data of the Whitehall II sub‐study” (MSD‐IDREC‐C1‐2011‐71).

*Inclusion- & exclusion criteria*

Participants were not eligible if they had MRI contraindications, a history of dementia or neurological illness, displayed significant abnormalities on structural MRI scans, had a history of major depressive disorder (assessed using the Structured Clinical Interview for DSM-IV mood disorders (SCID)), reported use of anti-depressant or other psychotropic medication, or were unable to travel to Oxford without assistance.

*Depressive symptoms*

Depressive symptoms were assessed using the 20-item Center for Epidemiological Studies-Depression Scale (CES-D).

*MRI measures*

Imaging data was acquired using a Verio Siemens 3.0 Tesla scanner (TR: 2,530 ms, TE: 1.79/3.65/5.51/7.37 ms, TI: 1,380 ms, flip angle: 7°, slice thickness: 1.0 mm, FOV: 256 × 256 mm) and a Prisma Siemens 3.0 Tesla scanner (TR: 1,900 ms, TE: 3.97 ms, TI: 904 ms, flip angle: 8°, slice thickness: 1.0 mm, FOV: 192 × 192 mm).

*Key references*

- Filippini et al., Study protocol: the Whitehall II imaging sub‐study. BMC Psychiatry, 2014, 14:159. Doi:10.1186/1471‐244X‐14‐159

## **Patient-control cohorts**

### **MOTAR**

*Sample source*

MOod Treatment with Antidepressant or Running (MOTAR), intervention study by VU medical center, Amsterdam, the Netherlands.

*Population*

Participants with current depressive disorders, current anxiety disorder (assessed using the CIDI), and healthy controls, participating to a treatment study recruited from specialized mental health care centers (patients) and general population (healthy controls).

*General description & procedures*

Baseline data for MOTAR was collected between 2012 and 2019. The MOTAR study is an intervention study aiming to examine the effectivity of 1) running therapy compared to 2) treatment with antidepressant medication over 16 weeks. Depressive and/or anxiety disorder diagnosis was assessed using the CIDI. A subgroup of the participants is asked to participate in the neuroimaging substudy (N=123). Only baseline measurements were included in the current study. All participants provided written consent and the study was approved by the Medical Ethical Committee VU University Medical Centre.

*Inclusion- & exclusion criteria*

Exclusion criteria included antidepressant use in the past two weeks, use of psychotropic medication, regular exercising (more than once a week) or medical contra-indications to running therapy or antidepressants, primary psychiatric diagnosis other than MDD or anxiety disorders, acute suicide risk, medical contraindications to running therapy or antidepressants (e.g. heart disease), MRI contraindications and a present or past internal or neurological disorder.

*Depressive symptoms*

Depressive symptoms were assessed using the 30-item Inventory of Depressive Symptomatology - Self Report (IDS-SR).

*MRI measures*

Imaging data was acquired using a Philips 3.0 Tesla scanner with the following parameters: TR: 9 ms; TE: 3.5 ms; slice thickness: 1 mm, FOV: 256 x 256 mm, 170 slices.

*Key references*

- Lever-van Milligen, B.A., Verhoeven, J.E., Schmaal, L. et al. The impact of depression and anxiety treatment on biological aging and metabolic stress: study protocol of the Mood treatment with antidepressants or running (MOTAR) study. BMC Psychiatry 19, 425 (2019). https://doi.org/10.1186/s12888-019-2404-

### **NESDA**

*Sample source*

NEtherlands Study of Depression and Anxiety (NESDA), multi-center study by VU medical center, Amsterdam, Leiden University, Leiden and Rijksuniversiteit Groningen, Groningen, the Netherlands.

*Population*

Participants with current depressive disorders, current anxiety disorder (both assessed using the CIDI), and healthy controls, recruited from the general community (healthy controls), general practitioners and specialized mental health care centers.

*General description & procedures*

NESDA is a large-scale, multisite, longitudinal, observational cohort study. Of the 2981 NESDA respondents, a subgroup of participants was asked to participate in the NESDA neuroimaging study (n=301). Participants were eligible to participate in the neuroimaging study if they were aged between 18 and 57 years, met the DSM-IV criteria for diagnosis of major depressive disorder (MDD) and/or an anxiety disorder (panic disorder, social anxiety disorder, and/or generalized anxiety disorder) in the 6 months preceding the baseline interview, or had no lifetime DSM-IV diagnosis (i.e. healthy controls). Diagnoses according to DSM-IV algorithms were established using the structured Composite International Diagnostic Interview, lifetime version 2.1, administered by a trained interviewer. Participants underwent magnetic resonance imaging in one of the three participating centers: Leiden University Medical Center, Amsterdam Medical Center, and University Medical Center Groningen. Scanning took place between 2005 and 2007. The ethical review boards of each center approved this study. All participants provided written informed consent after receiving written information. Only baseline measurements were included in the current study.

*Inclusion/ exclusion criteria*

NESDA neuroimaging exclusion criteria for patients were the presence of axis-I disorders other than MDD, panic disorder, social anxiety disorder, and/or generalized anxiety disorder and any use of psychotropic medication other than stable use of SSRIs or infrequent benzodiazepine use (i.e. equivalent to 2 doses of 10 mg of oxazepam 3 times per week or use within 48 hours prior to scanning). NESDA neuroimaging exclusion criteria for all participants were the presence or history of major internal or neurological disorders, dependence on or recent abuse (past year) of alcohol and/or drugs, hypertension, and general magnetic resonance imaging contraindications. Healthy controls were currently free of, and had never met criteria for, depressive or anxiety disorders or any other axis-I disorder and were not taking any psychotropic medication. Of the imaging participants, 12 were excluded due to bad image quality, one because the interval between baseline interview and scan was too long and two did not complete the scan due to claustrophobia, reducing the final sample to 286.

*Depressive symptoms*

Depressive symptoms were assessed using the 30-item Inventory of Depressive Symptomatology - Self Report (IDS-SR).

*MRI measures*

Imaging data was acquired using three Philips 3.0 Tesla scanners with the following parameters: TR: 9 ms; TE: 3.5 ms; slice thickness: 1 mm, FOV: 256 x 256 mm, 170 slices.

*Key references*

- Penninx, B. W. J. H., Beekman, A. T. F., Smit, J. H., Zitman, F. G., Noeln, W. A., Spinhoven, P., … & Van Dyck, R. (2008). The Netherlands Study of Depression and Anxiety (NESDA): rationale, objectives and methods. International Journal of Methods in Psychiatric Research, 17(3), 121–140. doi:10.1002/mpr.256
- Penninx, B. W. J. H., Eikelenboom, M., Giltay, E. J., Van Hemert, A. M., Riese, H., Schoevers, R. A., Beekman, A. T. F. (2021). Cohort profile of the longitudinal Netherlands Study of Depression and Anxiety (NESDA) on etiology, course and consequences of depressive and anxiety disorders. Journal of Affective Disorders 287, 69–77. doi: 10.1016/j.jad.2021.03.026
